# Supplementary material for: Co-option of neurotransmitter signaling for inter-organismal communication in C. elegans
Source: Nat Commun. 2019 Jul 18;10:3186. doi: 10.1038/s41467-019-11240-7 (PMC6639374; doi:10.1038/s41467-019-11240-7)
Supplement: Supplementary file 1 — Supplementary Information [file 41467_2019_11240_MOESM1_ESM.pdf]

Co-option of neurotransmitter signaling for inter-organismal communication in *C. elegans*

Chute et al.

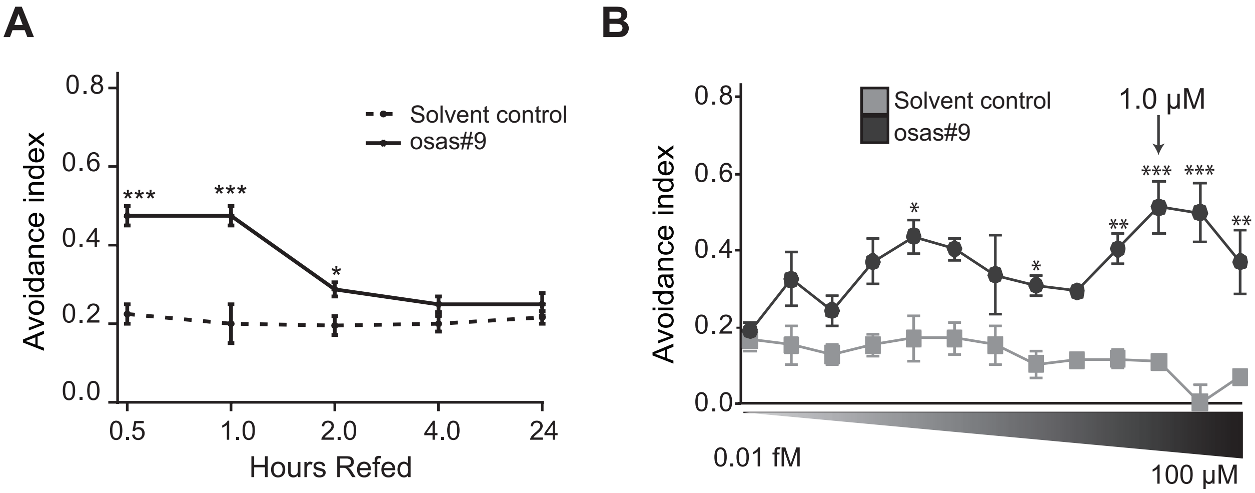

Supplementary Figure 1. A) Attenuation of osas#9 avoidance response by *E. coli* OP50. Animals reintroduced to *E. coli* OP50 for two hours exhibited an reduced avoidance response to osas#9, with complete attenuation at four hours, n ≥ 3 trials. **B)** osas#9 exhibits avoidance response over a broad range of concentrations (fM - μM) in YA wild type animals, n ≥ 3 trials. Data presented as mean ± S.E.M; * $p < 0.05$, ** $p < 0.01$, *** $p < 0.001$, one factor ANOVA with Sidak's multiple comparison posttest. Asterisks displayed without bar depict compared osas#9 avoidance to respective solvent control within groups.

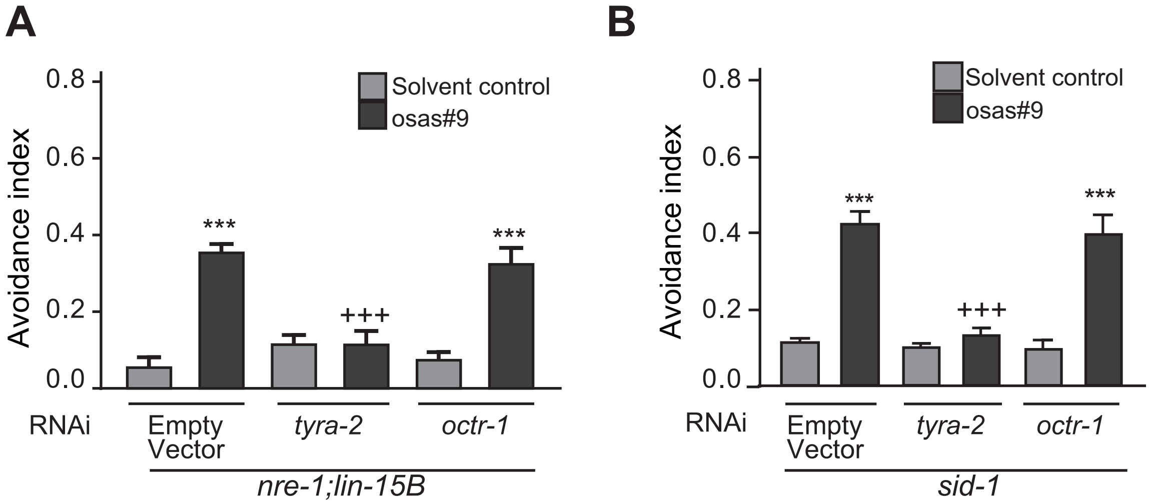

Supplementary Figure 2. A-B) *tyra-2* RNAi knockdown results in loss of avoidance to osas#9. Animals cultured at 15 °C and fed *tyra-2* RNAi clones were defective in response to osas#9 in two different RNAi sensitive backgrounds **A)** *nre-1(hd20);lin-15B(hd126).* n ≥ 10. **B)** *sid-1(pk3321).* n ≥ 3. Data presented as mean ± S.E.M; *** $p < 0.001$. Asterisks depict comparison between test solution and respective solvent control. '+' signs represent same p-value as asterisks, but representing difference between response of a strain/conditions in comparison to wild type.

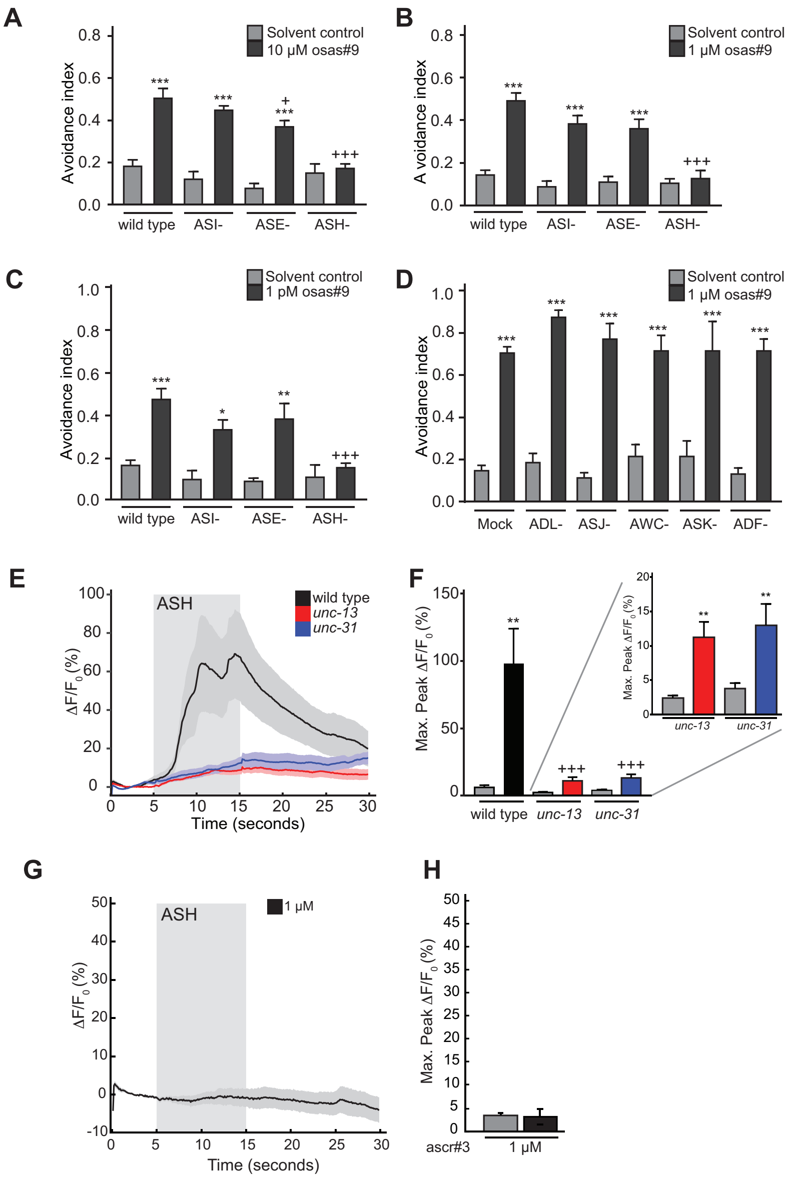

Supplementary Figure 3. Role of different sensory neurons in osas#9 avoidance behavior. **A-C)** Genetically ablated ASH, ASI, and ASE neurons were tested for their response to various concentration of osas#9: **A)** 10 μM, **B)** 1 μM, and **C)** 1 pM. n ≥ 3 trials. **D)** Sensory neurons not required for osas#9 avoidance. Note that ADL is not required for osas#9 avoidance. All ablated animals were tested with at least 10 animals with the exception of ADF, for which 7 animals were tested. **E,F)** ASH neurons exhibit significant calcium transients upon 1 μM osas#9 stimulation in both *unc-13* and *unc-31* mutant background. However, the dynamics of the calcium change in both mutant backgrounds are distinct from wild type animals in both magnitude and OFF response upon stimulus removal. **G)** ASH calcium transients are osas#9 specific. Exposure to an unrelated ascaroside, 1 μM ascr#3 (black), does not elicit calcium transients in the ASH neuron (exposure, gray-shaded region), n = 19 animals. **H)** Maximum fluorescence intensity in transgenic worms before (solvent control) and during exposure to 1 μM ascr#3. Data presented as mean ± S.E.M; * $p < 0.05$, ** $p < 0.01$, *** $p < 0.001$, one factor ANOVA with Sidak's multiple comparison posttest.

Asterisks depict comparison between test solution and respective solvent control. '+' signs represent same p value as asterisks but representing difference between response of a strain/conditions in comparison to wild type.

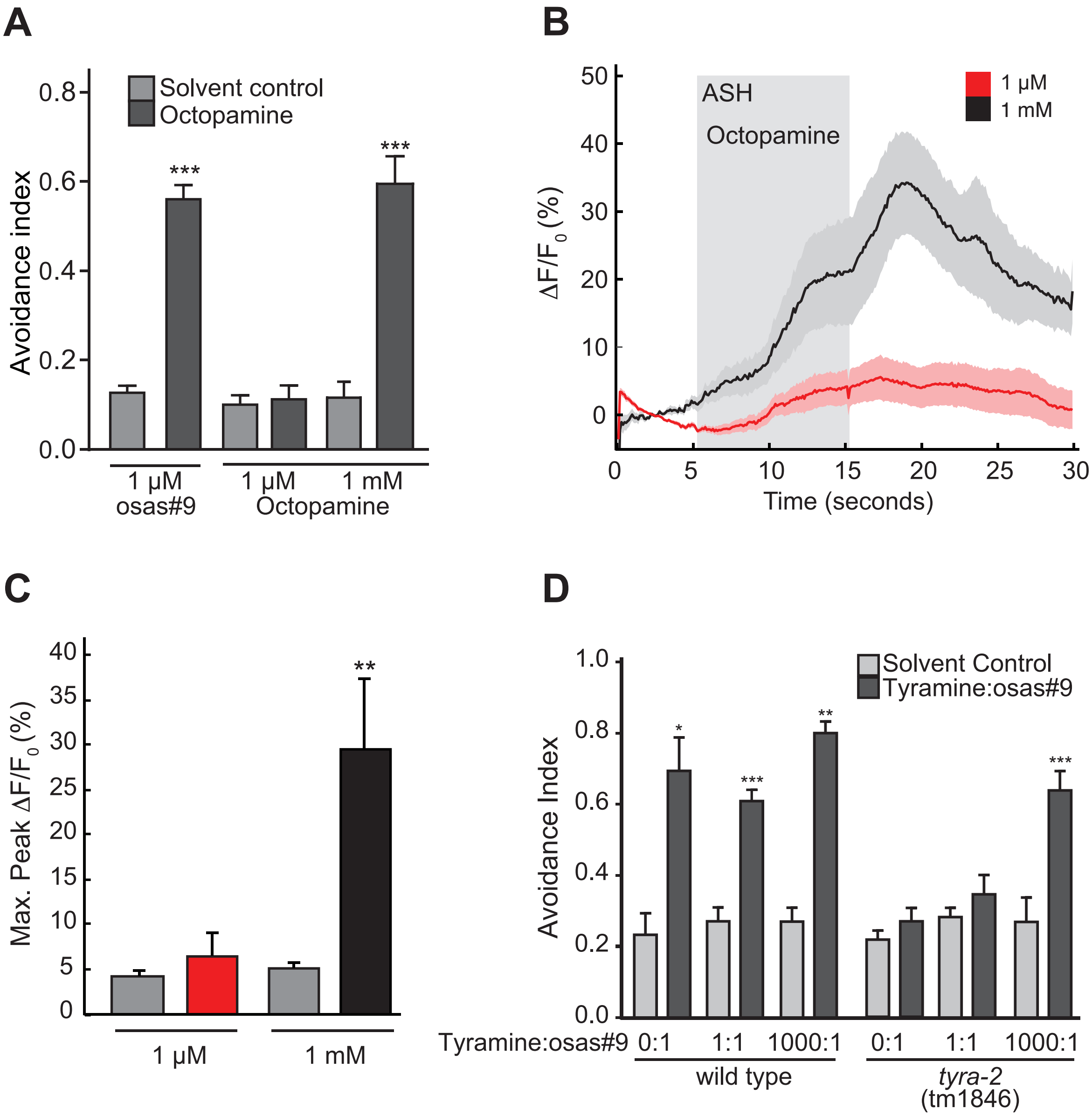

Supplementary Figure 4. Octopamine elicits avoidance at high concentrations. **A)** Animals do not avoid 1 μM octopamine but does avoid 1 mM octopamine. n ≥ 3 trials. **B)** Calcium dynamics in ASH sensory neurons upon exposure (gray-shaded region) to 1 μM (red) and 1 M (black) octopamine. n ≥ 10 animals. **C)** Maximum fluorescence intensity in transgenic worms before (solvent control) and during exposure to octopamine. **D)** Wild type and *tyra-2*(tm1846) animals were exposed to mixtures of tyramine and osas#9. In each mixture, the concentration of osas#9 was consistent at 1 μM. Tyramine was added in increasing rations of 0:1 (no tyramine), 1:1 (1 μM tyramine), and 1000:1 (1 mM tyramine). Wild type animals avoided every ratio mixture of cues, while *tyra-2*(tm1846) animals only avoided 1000:1. N = 5 trials, Tyramine:osas#9 avoidances were compared against paired solvent control values via a Student's t-test. Data presented as mean ± S.E.M; * $p < 0.05$, ** $p < 0.01$, *** $p < 0.001$, Student's t-test. Asterisks depict compared solution of interest to the solvent control.

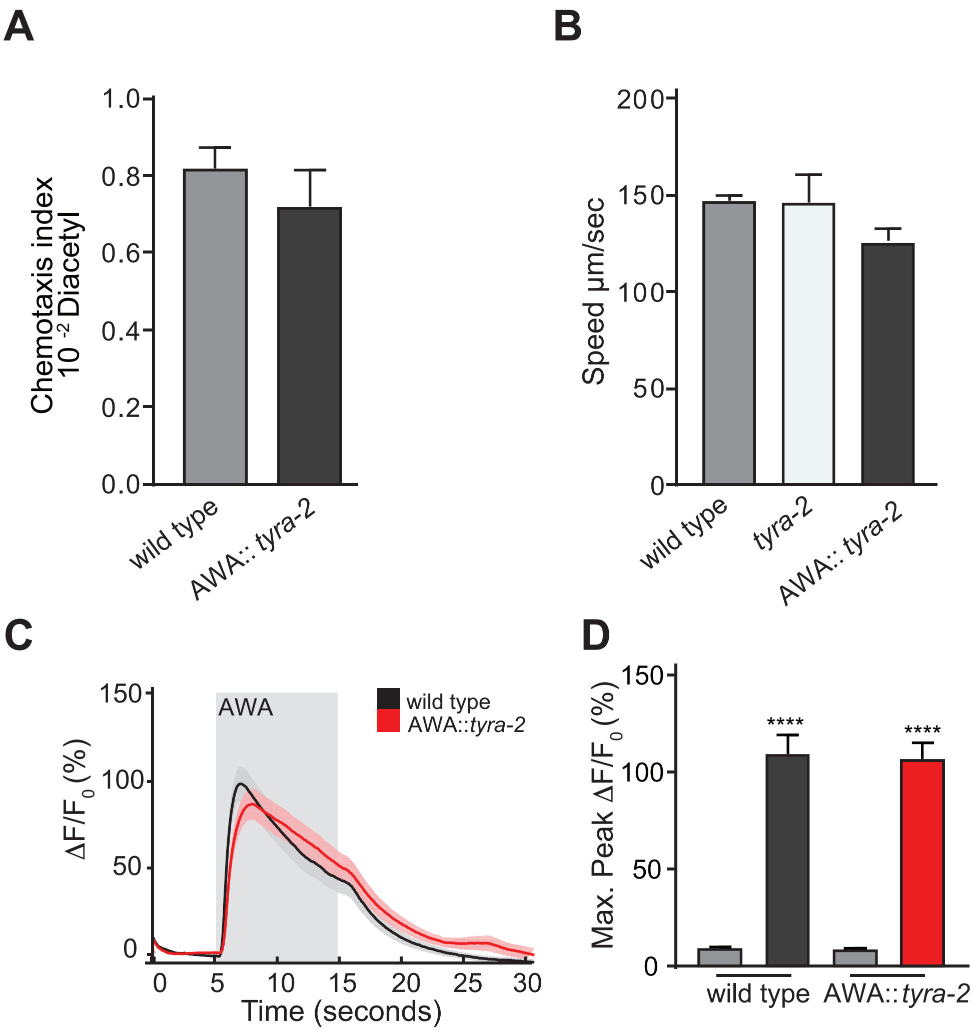

Supplementary Figure 5. Ectopic expression of *tyra-2* in AWA neurons does not affect AWA-specific behaviors. **A)** Chemotaxis to 10^{-2} diacetyl was unaffected by AWA::*tyra-2.* n ≥ 7. **B)** Locomotory behaviors were unaltered in AWA::*tyra-2* animals. Wild type, *tyra-2 lof*, and AWA::*tyra-2* speeds are not statistically different. n ≥ 3 trials. Data presented as mean ± S.E.M. Panel S6A, Student's t-test, Panel S6B, one factor ANOVA with Sidak's multiple comparison posttest. **C)** AWA::GCaMP2.2b animals (black) and AWA::*tyra-2*::GCaMP2.2b animals (red) show depolarization when exposed to 10^{-2} diacetyl in a microfluidic olfactory chip. Shaded gray region depicts time when animals were subjected to the stimulus, n≥8 animals. **D)** Maximum peak fluorescence before (solvent control) and during exposure to 10^{-2} diacetyl. Data presented as mean ± S.E.M; *P<0.05, **P<0.01, ****P<0.0001. Asterisks displayed without bar depict compared osas#9 avoidance response to the solvent control.

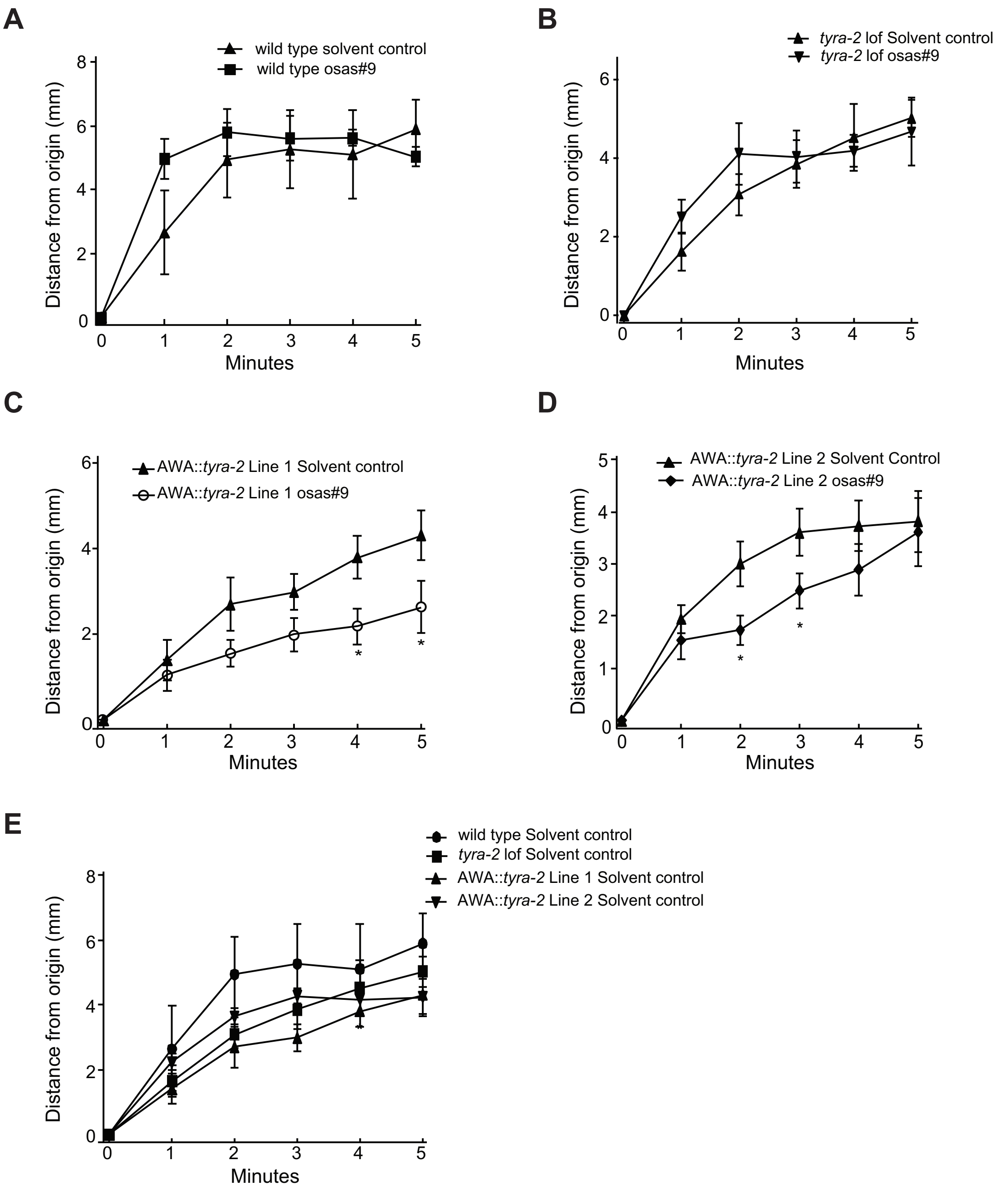

Supplementary Figure 6. Leaving rates for animals expressing *tyra-2* ectopically in AWA neurons are slower than both wild type and *tyra-2 lof* animals at 10 pM osas#9. **A)** Wild type. n ≥ 3 trials. **B)** *tyra-2*. n = 6 trials. **C,D)** Two different lines of AWA::*tyra-2* display slower leaving rates at 10 pM osas#9. n ≥ 6 trials Line 1, and n ≥ 7 trials Line 2. **E)** Comparison of solvent control for all strains in leaving assay. None of the animals varied in their response. n ≥ 3 trials. Data presented as mean ± S.E.M; * $p < 0.05$, one factor ANOVA with Sidak's multiple comparison posttest.

Source	Strain	Genotype (allele)	Avoid osas#9?
CGC	CB1489	*him-8*(e1489)	yes
Komuniecki	FX01846	*tyra-2* (tm1846)	no
Komuniecki	OH313	*ser-2* (pk1357)	yes
Suo	VN280	*ser-6* (2146)	yes
Alkema	QW569	*octr-1* (ok371)	yes
Komuniecki	DA1774	*ser-3* (ad1774)	yes
Chase	LX702	*dop-2* (vs105)	yes
Chase	LX703	*dop-3* (vs106)	yes
Alkema	QW284	*tdc-1*(n3420)	yes
Alkema	CX11839	*tyra-3* (ok325)	yes
Ambros	NL3321	*sid-1* (pk3321)	yes
Sternberg	PT839	*osm-9(ky10); him-5(e1490)*	no
Bargmann	CX7265	*osm-9*(ky10) IV;yzEx53 [*osm-10*::*osm-9*, *elt-2*::gfp]	yes
Srinivasan	JSR19	*tyra-2* (tm1846);worEx12[pLR306_p*nhr-79_tyra-2*]	yes
Srinivasan	JSR23	N2;worEx13[p*tyra-2*::*tyra-2*::GFP]	n.d.
Alkema	QW1853	*tyra-2* (tm1846);worEx14[p*tyra-2*::*tyra-2*::GFP @ 1ng/µL]	yes
Srinivasan	JSR45	*tyra-2* (tm1846);worEx15[pLR305_p*odr-10_tyra-2*]	no
Srinivasan	JSR47	*tyra-2* (tm1846);worEx15[pLR305_p*odr-10_tyra-2*]	no
Srinivasan	JSR50	*tyra-2* (tm1846) ;KyEx2865 [p*sra-6*::GCAMP3 @ 100 ng/µL])	n.d.
Srinivasan	JSR51	JSR45;kyIs598 [*gpa-6*::GCaMP2.2b 50 ng/µL]	n.d.
Srinivasan	JSR100	*tyra-2* (tm1846);KyEx2865 [p*sra-6*::GCAMP3 @ 100 ng/µL])	n.d.
Srinivasan	JSR104	JSR100;(*tyra-2* (tm1846);worEx12[pLR306_pnhr-79_tyra-2]	n.d.
Srinivasan	JSR105	*unc-13*(e51);KyEx2865[p*sra-6*::GCAMP3]	n.d.
Srinivasan	JSR106	*unc-31*(e928);KyEx2865[p*sra-6*::GCAMP3]	n.d.
Bargmann	CX10979	N2;KyEx2865 [p*sra-6*::GCAMP3 @ 100 ng/µL])	n.d.
Albrecht	CX14887	N2;kyIs598 [*gpa-6*::GCaMP2.2b 50 ng/µL]	n.d.

Supplementary Table 1. List of Strains used in this study

Source	Construct	Notes
Fire vector kit	pPD95_75	GFP
Rene Garcia	pLR305	destination vector
Rene Garcia	pLR306	destination vector
Rene Garcia	pLR304	destination vector
Srinivasan	JSR#CDC8	*gpa-6* entry clone
Srinivasan	JSR#CDC9	ASH::*GPA-6*
Srinivasan	JSR#CDC2	*tyra-2* entry clone
Srinivasan	JSR#CDC1	p*nhr-79* entry clone
Srinivasan	JSR#CDC4	p*odr-10* entry clone
Srinivasan	JSR#CDC3	ASH::TYRA-2
Srinivasan	JSR#CDC6	AWA::TYRA-2
Srinivasan	JSR#CDC24	p*gpa-6::gpa-6::RFP::unc-54*
Vidal Library	F01E11.5	*tyra-2* RNAi clone
Vidal Library	F14D12.6	*octr-1* RNAi clone
Vidal Library	pL4440	Empty RNAi clone

Supplementary Table 2. List of Plasmids used in this study

Purpose	Gene	Primer	Sequence
tyra-2 ASH rescue construct	*tyra-2* (from genomic)	F	attB5*ggcttatccgttgtggagaa*
		R	attB2*ttggcccttccttttctctt*
tyra-2 ASH rescue	*pnhr-79* (from genomic)	F	attB1*gtgcaatgcatggaaaattg*
		R	attB5r*atacacttcccacgcaccat*
tyra-2 translational fusion (PCR)	*tyra-2* (from genomic)	F	*atgttttcacaagtttcaccaca*
		F nested	*ttcacaagtttcaccacattacaa*
		R (w overhang in caps)	*AGTCGACCTGCAGGCATGCAAGCT gacacgagaagttgagctgggtttc*
	GFP (pPD95_75)	as found on wormbook chapter: reporter gene fusion	
AWA *tyra-2* misexpression	*podr-10* (from genomic)	F	attB1*ctcgctaaccactcggtcat*
		R	attB5r*gtcaactagggtaatccacaattc*
RT-qPCR	*ama-1*	F	GGAGATTAAACGCATGTCAGTG
		R	ATGTCATGCATCTTCCACGA
	tyra-2	F	GAGGAGGAAGAAGATAGCGAAAG
		R	TGTGATCATCTCGCTTTTCA
gpa-6 ASH rescue	*gpa-6* (from genomic)	F	attB5 cgtctctttcgtttcaggtgtat
		R	attB2 tattttcaaagcgaaacaaaaa
gpa-6 translational fusion (plasmid)	p*gpa-6::gpa-6* (from genomic)	F	acatctggtacccctcaatttcccacgatct
		R	acatctaccggtctcatgtaatccagcagacc
	unc-122::RFP	F	acatctaccggtATGGTGCGCTCCTCCAAG
		R	ttaataggtaccTGGTCATAGCTGTTTCCTGTG

Supplementary Table 3. List of Primers used in this study
